# The Editor's Letter-Box

**Published:** 1914-04-25

**Authors:** 


### THE EDITOR'S LETTER-BOX.

### Non-Panel Men and the "Medical Directory."

*To the Editor of* The Hospital.

Sir,—The suggestion put forward by Dr. Nicholls in your issue of April 18, to the effect that panellers and non-panellers should be separable from one another by some distinguishing mark or sign in the *Medical Directory*, is one which he will have to settle with the proprietors of that annual. Personally, I doubt whether he will succeed in the endeavour; but by all means let him try. What I wish to point out is that his alternative suggestion, that if the proprietors of the *Medical Directory* declined to entertain the idea, it should be carried out in spite of them by not returning their annual circular and so incurring an asterisk before one's name, is quite impracticable. In the first place, a great many panellers would, through carelessness or forgetfulness, also incur the asterisk. And in the second, those non-panellers who adopted this way of indicating their refusal to join a panel would debar themselves permanently from altering or amplifying the *précis* of their professional careers which the *Medical Directory* contains. They would even be debarred from changing their address or their telephone number, save at the risk of being thought to be panellers!—Yours faithfully,

Practical.

